# The Promises and Pitfalls of Digital Technology in Its Application to Alcohol Treatment

**DOI:** 10.35946/arcr.v36.1.14

**Published:** 2014

**Authors:** Frederick Muench

**Affiliations:** Frederick Muench, Ph.D., is a clinical psychologist and the director of digital health interventions in the Department of Psychiatry at North Shore Long Island Jewish Health System in New York and an adjunct at New York University’s Interactive Telecommunications Program, New York.

**Keywords:** Alcohol use, abuse, and dependence, treatment, assessment, intervention, technology, digital technology, electronic health technology, computer technology, Internet, telecommunication, literature review

## Abstract

Individuals seeking to change their alcohol use form a heterogeneous group with varied treatment goals—including moderation and abstinence—that therefore requires flexible treatment options. The availability of alcohol in the United States, and the pervasive social pressure to drink, warrant treatments that support individuals outside the treatment environment and that foster coping and self-regulation in the face of these demands. Emerging digital technologies show promise for helping both to hone therapies to clients’ individual needs and to support clients in settings beyond the clinic. In the broader health care arena, digital health technologies (DHTs) are transforming how health professionals assess, prevent, and treat both physical and mental health problems. DHTs include assessments and interventions delivered via computer, Internet, mobile phone, and wireless or wearable device technologies. The emerging literature examining within-treatment and mobile DHTs highlights an opportunity to create personalized alcohol treatments for every person seeking care. Despite the promises DHTs may hold, however, there still are many potential risks to using them and a number of challenges regarding how to integrate them into treatment successfully. This article will review the current and potential advantages of DHTs in alcohol treatment and the technological, personal, organizational, and systemic limitations of integrating various technology-based assessment and intervention programs into care.

Using methods like the Network for the Improvement of Addiction Treatment (NIATx) that identify the strengths and weaknesses of current treatment processes (Karlsson et al. 2010), treatment providers can identify the components of care in which DHTs may have the most and least impact, as well as the obstacles that arise when attempting DHT integration. This article’s initial section will review some of the emerging trends and promises of DHTs inside and outside of alcohol treatment, including consumer-based DHTs, DHTs for treatment initiation and intake, DHTs to enhance alcohol treatment services and services for comorbid conditions, DHTs to extend care beyond the clinic and increase salience of the therapeutic environment, mobile assessment and just-in-time interventions, combining DHTs with in-person support, and finally DHT acceptability from the perspective of the client.

## The Promise of DHTs

### Direct-to-Consumer DHTs

Ample evidence shows that the majority of individuals who could benefit from alcohol treatment never seek care, suggesting a need to expand the reach and accessibility of treatment. Thus, the proliferation of DHTs that increase people’s awareness of how much they drink or their relationship with drinking could meet this need. To date, the development and implementation of digital health services marketed directly to consumers has largely occurred outside of the traditional professional in-person treatment community. These technologies use both brief, one-time computer-based screening and brief interventions and multimodular long-term Internet programs (see table) (Brendryen et al. 2013; Carey et al. 2009; Cunningham et al. 2011; Hester et al. 2013). Such self-guided interventions have typically targeted a lower-severity population than those who tend to seek treatment for alcohol use disorders (AUDs), extending the reach of available options for individuals along the broader problem-drinking spectrum. The rise of mobile-phone applications has produced a large number of alcohol-specific mobile programs available directly to the consumer outside of care, ranging from blood alcohol content (BAC) calculators to coping-skills programs. However, almost no research exists to support the efficacy of these applications beyond their face validity (Cohn et al. 2011). For individuals in recovery, there also are numerous Web-based programs and mobile applications such as online video support meetings, 12-step meeting-finder apps, virtual sponsors, and recovery coaches (Cohn et al. 2011). These applications are designed to make the recovery process more efficient by enhancing what individuals are already doing, such as going to meetings or connecting with those in recovery.

## Facilitating Treatment Initiation With DHTs

Although the actual integration of DHTs into alcohol treatment has been limited in comparison with the explosion of direct-to-consumer programs, DHTs specifically designed for implementation within traditional treatment are beginning to emerge (Carroll and Rounsaville 2010) in the research literature and offer significant promise to increase the efficiency and quality of care. In health care, and in other settings such as schools and workplaces, computer and mobile DHT screening and brief intervention programs have enhanced the ability to reach individuals opportunistically in those moments when they are motivated to seek more information about their drinking. For example, programs such as Hazelden, a large inpatient and outpatient treatment organization with centers in 5 states, include digital screeners on their Web sites to assist and engage individuals seeking services. Along similar lines, treatment program Web sites can include appointment schedulers for those who are reluctant to initiate help seeking with a phone call, as well as digital copies of all clinic forms (e.g., consent forms) to make the engagement process both more efficient and transparent. Ideally, these tools could be programmed to take into account a client’s insurance, financial, and location constraints as well as his or her treatment preferences (Boudreaux et al. 2009).

Evidence from the general mental health literature suggests that once individuals agree to start treatment, they often have misconceptions about the treatment experience. Educating clients about what to expect can improve retention and therapeutic outcomes. Technology-based educational orientations (i.e., orientation videos) can be a useful and efficient means to orient someone to the treatment process (Zwick and Attkisson 1985). Video orientations are as effective as in-person orientations for many health and behavior problems and improve overall outcomes compared with no-orientation control groups (Walitzer et al. 1999; Zwick and Attkisson 1985). Just as screening and feedback tools can be added to Web sites, pretreatment video orientations can be added to intakes to demystify the treatment process either before treatment entry or in the waiting room during the first appointment.

## DHTs to Improve Client Intake

Digital technologies also can almost certainly improve the burdensome client intake process, which at most treatment centers typically involves hours of paper-and-pencil questionnaires and forms that may or may not be transferred to an electronic health record (EHR). The benefits of digital assessments compared with both paper-and-pencil and faceto-face assessment have been repeatedly supported by a robust literature over the last 40 years (Paperny et al. 1990; Skinner and Allen 1983; Tourangeau and Smith 1996; Turner et al. 1998). Although digital assessments provide numerous benefits within the clinic from an administrative perspective (i.e., reduced staffing costs, improved compliance with reporting, etc.), several advantages stand out from a clinical perspective as well. Digital assessments can collect more relevant information from clients more efficiently using decision-support algorithms. These programs collect broad information through liberal stem questions then target the assessments to the most relevant domains, which can be missed sometimes during traditional assessments (Davenport et al. 1987; Paperny et al. 1990; Quaak et al. 1986).

A related body of research provides significant evidence that computer-assisted interviews can collect more sensitive information than face-to-face interviews (Weisband and Kiesler 1996), including data pertaining to alcohol use (Lucas et al. 1977), sexual behavior, and drug use (Skinner and Allen 1983; Tourangeau and Smith 1996; Turner et al. 1998). Recently, Kang and Gratch (2010) found that individuals seeking treatment for social phobia revealed more information to a virtual avatar than a human counselor, revealing that client–DHT interactions may be beneficial during the intake process. Whereas the mechanisms behind this observation are not entirely clear, it seems that the fear of judgment and negative feedback that comes from revealing sensitive information to a human does not apply when disclosing to a digital system. This suggests that individuals perceive digital systems to be a safer means to disclose potentially stigmatizing information. Such findings imply that by integrating digital assessment into care, treatment providers can collect the most relevant data while also helping clients feel comfortable disclosing personal information that they might not disclose otherwise. Collecting more sensitive and relevant information more efficiently in turn promotes more informed treatment suggestions and diagnoses (Bennett and Hauser 2013).

Similar to medical systems like ISABEL (Ramnarayan et al. 2004), which can identify a symptom cluster as being a strong indicator of a specific diagnosis, computerized feedback systems can help providers develop tailored treatment plans driven by these more precise assessments. As described below, they can also trigger use of standalone DHTs to mimic current therapies or augment treatment by offering interventions in domains outside staff expertise (e.g., HIV risk reduction) (Litvin et al. 2013).

## Enhancing Care Using DHTs

The enhancement or partial replacement model of digital interventions, in which a DHT delivers all or a portion of care that would traditionally be delivered by a counselor, is designed to deliver standardized empirically supported therapies electronically to enhance the services offered within a clinic. This model has been shown to be as or more effective than stand-alone in-person care in several studies of poly-substance users (Litvin et al. 2013; Marsch et al. 2014). For example, Carroll and colleagues (2009, 2010) integrated a digital cognitive behavioral therapy (CBT) intervention into outpatient substance abuse treatment and saw a significant reduction in the number of positive urine samples and an increase in participants’ use of coping skills when compared with treatment as usual. Similar results have been found in studies of other systems such as the Therapeutic Education System (TES) when tested with polysubstance users (Marsch et al. 2014). These programs offer multiple modules that, like in-person interventions, can be delivered flexibly and tailored to the individual. It is important to note that these studies on enhancement DHTs have been conducted within formal treatment settings where there is significant in-person therapeutic support to encourage use and respond to questions about the DHT. Studies have revealed that without therapist contact, multiple-session Internet-based interventions are rarely used after a few sessions (Cunningham et al. 2011), which should be noted when interpreting results of these studies. Another enhancement to care that typically involves more clinician oversight is using new technologies rarely integrated into traditional care, such as virtual-reality cue exposure for alcohol use (Lee et al. 2007). Virtual-reality programs expose individuals to virtual alcohol-related cues and teach them coping skills through modeling and rehearsal. These technologies are examples of how DHTs expand what currently is possible within a single treatment setting.

## Addressing Comorbidities Through DHTs

Individuals with AUDs often present with multiple comorbid problems, and difficulties arise for treatment programs in creating a continuum of care when certain conditions lie outside a clinic’s area of expertise. Although clinicians cannot be trained in empirically supported treatments for all disorders, DHTs can augment care for individuals with specific pressing needs that are related, but secondary, to their alcohol use, such as HIV risk reduction and polysubstance abuse (Moore et al. 2013). The proliferation of DHTs across health domains beyond substance abuse treatment (Lal and Adair 2014; Portnoy et al. 2008) could improve the likelihood that comorbid conditions can be addressed in a single treatment setting. In those areas where treatment programs do not have the expertise and budgets to meet the diverse needs of treatment seekers, DHTs can help deliver specialized treatment for a number of conditions such as insomnia and depression without the need for significant staff expertise in the relevant health domains. Those who need more intensive services for comorbid disorders can be referred to specialty care.

## Extending Care Beyond the Clinic

Most of the benefits of DHTs described above can be implemented via any digital device—computer, tablet, or phone—but only mobile and wireless devices expand the use of DHTs into a patient’s everyday experience. Mobile DHTs are uniquely capable of reaching, assessing, and intervening with individuals in their natural environment over extended periods to provide just-in-time therapeutic support and salience beyond the clinic. These tools can keep individuals engaged in care (Branson et al. 2013) and facilitate long-term continuing-care contact (Gustafson et al. 2014).

### Improving Treatment Attendance

In the last 10 years, DHT research outside of alcohol treatment has demonstrated that DHTs, particularly those disseminated via mobile messaging and e-mail, increase appointment adherence in medical settings (Gurol-Urganci et al. 2013). Although the evidence is mixed as to whether a text message or e-mail is any better than a phone call for increasing appointment adherence, digital messaging has advantages over phone calls. It reduces staffing costs and increases efficiency, because multiple messages can be programmed simultaneously and responses can be collected automatically and reviewed in a single sitting (Gurol-Urganci et al. 2013; Perron et al. 2013). From a client perspective, text or e-mail communications allow for increased confidentiality. Individuals do not have to speak out loud on their phones, can use their devices’ security settings to safeguard messages (Pal 2003), and can refer back to a message at any point after they have received it. E-mails also do not require the phone to be active when the message is sent, making clients less vulnerable to missed communications (Anhoj and Moldrup 2004).

### Therapeutic Salience

Even when clients engage regularly in treatment, they may fail to remain mindful of treatment goals and practices when outside the clinic. DHTs that remind clients of their goals could help them adhere to them in challenging situations. Because mobile messaging has become the most widely available mobile technology of the last 15 years, it has the largest empirical base at this time compared with smartphone applications. Numerous studies have shown that mobile messaging, including interactive voice response for substance users (Moore et al. 2013), can improve outcomes across physical and mental health disorders (Free et al. 2013). Some small mobile-messaging studies have been performed with problem drinkers (Suffoletto et al. 2012; Weitzel et al. 2007). Weitzel and colleagues (2007), for example, found that heavy-drinking college students not seeking treatment who received tailored mobile messages about drinking consequences via a personal digital assistant reported consuming fewer drinks per drinking day than a control group not receiving messages.

Adherence to the use of computer-based DHTs outside of the treatment setting tends to decline over time, even when traditional substance abuse treatment protocols are followed (Klein et al. 2012). Recent reviews suggest that the addition of mobile messaging and other prompts improves the effects of Web-based interventions, because they promote user action and engagement in the intervention (Fry and Neff 2009; Riley et al. 2011; Webb et al. 2010), which could be useful to trigger greater use of DHTs for alcohol and drug use that have low adherence (Brendryen et al. 2013; Cunningham et al 2009).

### Using Mobile Assessment Throughout Treatment

Computer-based assessments conducted in clinical settings during intake procedures can assess usual drinking times and trigger assessments and interventions when these moments occur. Following intake, real-time assessments administered via mobile and wireless devices can take the assessment process a step further by generating a record of the everyday experiences of clients in the real world (Hufford et al. 2002; Shiffman 2009). For example, Kuerbis and colleagues (2013) revealed that self-efficacy judgments collected via mobile assessment during the first week of treatment significantly predicted reduced problem drinking compared with static baseline assessments. Although it is beyond the scope of this paper to review the range of mobile assessments, they represent an opportunity to understand clients in their natural environment across subjective states, such as craving to drink and confidence to abstain or moderate (Shiffman 2009), while measuring objective parameters using context and location sensing (Vahabzadeh et al. 2010) and transdermal sensing (Hawthorne and Wojcik 2006). For example, Gustfason and colleagues (2014) used self-report items to measure subjective craving states while simultaneously collecting geolocation data to better understand craving in the context of the participant’s location. Other methods used in general health behavior change, such as qualitative journaling and ecological video journaling (Melton et al. 2013) and a range of data visualization dashboards, also provide new DHT methods to help providers understand their clients’ everyday lives.

## Just-in-Time Adaptive Interventions

Assessing the everyday experiences of clients can both provide a means to understand how they progress through treatment and also trigger personalized stepped care via just-in-time adaptive interventions (JITAIs) (Riley et al. 2011). Most adjunctive DHTs used at treatment facilities can flexibly adapt and tailor content as the individual progresses through care (Marsch and Gustafson 2013). However, newer mobile interventions are able to assess progress and adapt intervention timing, content, and strength in one’s environment based on changes in key outcome variables, a capability akin to that of phone-based stepped care interventions for AUDs (McKay 2009).

The most comprehensive mobile JITAI for substance use disorders is the A-CHESS system developed by Gustafson and colleagues (2011, 2014), which is designed to assess and support individuals continually following alcohol treatment. The program provides extended skills training over time based on a client’s current needs and a panic button for high-risk situations. It evaluates individuals for relapse risk based on their assessment results and/or via geolocation if they are entering a high-risk environment. Based on risk, it first sends the client reminders of his or her therapeutic goals, self-modeling audio and image reminders, and tailored educational and therapeutic materials. The application then triggers in-person peer and provider support if the individual is nonresponsive or requests additional assistance. When compared with a treatment-as-usual aftercare condition, the A-CHESS mobile application significantly reduced the number of risky drinking days and increased the number of abstinent days in an AUD sample over the 8 months following inpatient alcohol treatment. Moreover, these results were maintained for 4 months after participants stopped using the application, demonstrating that the A-CHESS system did not engender unhealthy dependence among participants upon the DHT.

Although research is in its nascent stages, soon many programs will work like A-CHESS, using subjective and objective parameters and a range of assessment tools to trigger JITAIs that help individuals either become aware of risky patterns that could lead to relapse or alert support networks in an emergency (Chih et al. 2014). A range of DHTs also offers the opportunity for providers to reach out to clients proactively for check-ins in addition to having client self-assessments drive care. Whereas client self-assessments may be more predictive of relapse and outcomes than those of counselors (Walton et al. 2000), both can reveal valuable information, and combining both types of assessment will help identify the best methods to monitor outcomes.

## The Power of Connection

One of the strongest methods for improving outcomes of AUD interventions seems to be the combination of DHTs with human support (Andersson and Cuijpers 2009; Christensen et al. 2009; Spek et al. 2007). As Fox (2013) suggests, the clinical value of technology lies not in its computing power but in its ability to connect providers to their patients. Although some evidence indicates that brief interventions through DHTs for low-severity populations may not require in-person contact (Cunningham et al. 2011), this finding has not been established in the cases of more severely affected populations needing long-term continued-contact interventions. The lack of both research on and evidence of efficacy of standalone DHTs in more severely affected populations underscores both the ethical concerns related to using DHTs in more severely affected populations without provider contact or guidance and the limitations of DHTs in general.

Overall, DHTs such as computer-based interventions without provider accountability or proactive alerts have extremely high attrition rates (Price et al 2012). This has been termed the “law of attrition” for Web-based interventions (Eysenbach 2005). As Postel and colleagues (2011) highlight, completion rates for Web-based alcohol intervention studies range from about 16.5 percent to 92 percent, depending on the study design, but are lower for real-world trials. For example, in the real-world trial of a Web-based computer continuing-care intervention, 90 percent of all individuals did not access the Web site after 6 months (Klein et al. 2012). Similarly, in the author’s automated text-messaging study to improve attendance in methadone treatment, clients responded to automated messages for the first couple of weeks even though they were told it was a completely automated system (Muench et al. 2012). When they received no response, they stopped texting. Mohr and colleages (2011) stress the importance of human accountability in technology-based interventions because of the demotivating nature of automated human–computer interaction over the long term and ethical concerns related to automated systems for high-severity populations. Fortunately, newer mobile interventions within and outside the alcohol treatment field, including prompts, JITAIs, and human support, seem to be resulting in more engagement than older, primarily Web-based interventions (Alemi et al. 1996; Fry and Neff 2009; Gustafson et al. 2014), highlighting the power of combining DHTs with provider support.

Even the direct-to-consumer market seems to recognize the value of person-to-person contact. Many consumer-based substance abuse DHTs for individuals in recovery connect users to 12-step groups or peer support rather than being standalone behavior-change support applications. Social media sites and discussion boards offer some social interaction to combat the loneliness that often is associated with the behavior change process and may be particularly helpful early on. Applications such as In The Rooms provide online 12-step groups, counteracting the justification that meetings are too far away or inconvenient. Alternative self-help programs such as SMART Recovery and Moderation Management offer online support meetings, expanding their reach to those regions in which only 12-step meetings are available in person. These are just a few examples of how DHTs can facilitate support and connect users to peer-based recovery services as adjuncts to care.

## Client Acceptability

No matter how sophisticated and responsive, DHTs will only improve treatment if clients accept and use them. Evidence strongly supports the acceptability of alcohol and other substance use DHTs to clients, whether the technologies are delivered in the context of treatment or via a mobile intervention (Moore et al. 2011; Muench et al. 2013). This is even true outside the domain of alcohol use among the most disenfranchised clients with severe mental illness (Ben-Zeev 2012). DHTs also can expand participant treatment options, further increasing client satisfaction and improving client engagement with the treatment of their choice. For example, Hester and colleagues (2013) revealed that a subgroup of participants enrolled in a DHT with an online interactive support group component who only chose to use the noninteractive components of the DHT had drinking outcomes equivalent to those who opted to participate in the group component. DHTs thus offer treatment-seeking populations the flexibility to choose the components of an intervention they find most helpful and relevant to their needs.

## Pitfalls of DHTs

### Barriers to Integration

The previous sections reviewed the benefits of DHTs and their promise for improving overall outcomes. However, the field still is in the nascent stages of this paradigm shift, and integration of DHTs into care faces significant barriers. The cost of development is one of the most globally pressing concerns, but few feasible resolutions exist. Similarly, no DHTs for alcohol treatment and care are currently reimbursable, despite the costs of development, implementation, and maintenance. Thus, providers have limited incentives to embrace these new models. Some of the most common barriers to integration are reviewed below. These issues fall into six broad categories, including finding/developing and managing DHTs, data security and privacy, consent, usefulness and efficacy concerns, organizational integration, and client concerns.

### Integrating DHTs Into Practice and ManagingTheir Data

At present, no single technology framework securely supports all of the requisite features of DHTs for substance abuse treatment, from intake to charting to continuing care. Using multiple fragmented programs that each lack some necessary features can increase staff burden and result in the needless generation of overlapping data structures (i.e., using multiple platforms or methods to collect the same data in a single treatment setting). Some more comprehensive DHT support systems can be used as adjuncts to a treatment center’s existing structure (Brendryen et al. 2013). However, these systems also face barriers, including limitations on data privacy, data sharing, business associates’ agreements, and a lack of control over program modifications. However, these systems do represent a promising means to begin integrating DHTs into care at a low level of development burden.

Some individual organizations have created their own internal systems (e.g., Hazelden) that are customizable to their specific needs. However, this requires substantial up-front capital and entails additional maintenance costs. Developing internal or custom systems involves forming an interdisciplinary team that includes user experience and user interface designers; front-end and back-end developers; data managers and analysts; and privacy, content, and health technology experts. As in other instances of technology integration, creating DHTs that meet the needs of providers will probably require a mix of internal and external systems, and most treatment programs will integrate technology in piecemeal, flexible ways. Newer research studies are combining and testing multiple DHT modalities such as Web-based and mobile components (Brendryen et al. 2013), as well as within-treatment and mobile programs (e.g., TES & A-CHESS), to lay the groundwork for more comprehensive care systems.

One of the newer challenges arising from the development of comprehensive programs centers on the massive amounts of big data that are collected by these systems, particularly information collected by ecological momentary assessment (EMA) and passive continuous sensing through mobile and wireless devices. Although these data sources represent some of the most promising components of JITAIs, continuously collecting heart rate, for example, while inquiring about craving to train a smart passive-sensing system requires new methods of data cleaning and analysis (Shiffman 2013). Cleaning the data alone requires significant resources because of the poor data quality collected by sensor-based sources (Kumar et al. 2013) and the increased risk of missing self-report data that accompanies EMA (Shiffman 2013). Analytical methods such as Bayesian modeling (Chih et al. 2014), dynamic systems modeling (Timms et al. 2013), and mathematical modeling (Banks et al. 2014)—which are not traditionally used in data analysis of client progress—can help clarify the dynamic relationship between real-time data and the temporal unfolding of the behavior-change process. However, collecting and analyzing this type of data requires new expertise and interdisciplinary collaboration that has not yet been integrated into existing systems of care. Moreover, all of these new and promising methods of understanding clients through data are coupled with the greatest barrier to integrating technology into care—ensuring data security and privacy.

## Data Security and Privacy

Integrating technology into treatment poses progressively more significant challenges related to data privacy and confidentiality as data collection grows and moves beyond the confines of the clinic. (See also article by Arora in this issue.) The most secure means to integrate technology is to use digital programs such as intake and follow-up assessments within treatment centers, which typically are protected by powerful firewalls like those used in all hospitals. However, as data collection expands beyond the confines of the agency, significant issues arise for maintaining privacy and security (Luxton et al. 2012). New options apply when using any external communication, but given the relative novelty of mobile health technology, these are nebulous at best. For example, a treatment provider can use messaging encryption services, but these services require all clients to have specialized apps or software, and the only guarantees of security at present come from the companies’ own claims. Recently, a company designed to evaluate apps on efficacy and security had to suspend its operations because it was certifying apps with security limitations. Because security and privacy essentially are the kryptonite of most external communication applications, agencies should assess their communication needs and goals before deciding to implement a mobile DHT. Simply reminding patients of their appointments, for example, may require a different level of security than providing an actual intervention.

Despite these limitations, technology offers the ability to be creative in developing assessment and intervention protocols depending on treatment or research goals. In the author’s current National Institute on Alcohol Abuse and Alcoholism (NIAAA)-sponsored study testing various forms of mobile messaging to reduce problem drinking, participants who are concerned about the privacy or security of their messages can be placed in a condition in which they receive messages that make no explicit reference to alcohol or drinking. In this group, the focus is on general motivation salience to meet personal goals. It is entirely possible that interventions need not mention the disease state to be effective but can instead be tailored based on variables such as time and other processes such as self-efficacy and context. Other options include using generic self-monitoring applications to assess and intervene with participants, or viewing their progress on a shared dashboard or via mobile messaging/e-mails to trigger a Web-based portal that is password protected and secure. Establishing best practices for ensuring data security and privacy when using DHTs is a work in progress. Until secure external communication technologies are invented, clinicians need to be creative in how they work with client populations outside the clinic.

## Using Consent Effectively for DHTs

Incorporation of DHTs into care not only requires creativity and data security but also consent forms modified to include all the potential risks of using a digital platform. These additions can increase the length of consent forms by several pages, since they must cover every potential risk of using the technology. The risks associated with digital communications are numerous and vary based on the types of technology employed. DHT consent forms likely will need to include information such as the scope of digital communication, information communicated and method used, inherent privacy risks of communication, security and storage of communication, use of outside vendors who have access to communication, security of external vendor applications, procedures for lost provider devices, training clients on how to secure communication devices, lack of control over timing of digital communication, likelihood of missed communications, inappropriateness of digital communication as an emergency platform, recorded digital communication as part of the client’s health record, risk of misinterpretation in text-based communication (e.g., cryptic tone or context), possible charges incurred by the client, protection of agency and agency staff devices, and opt-out and help options. These are just some of the topics that a consent form might need to cover related to digital communication, but all possible scenarios should be explored when an agency plans to employ DHTs.

## Evaluating DHT Usefulness and Efficacy

At present, the empirical literature evaluating DHTs lags behind the number of DHTs developed for alcohol use and substance use more generally. The process of validating an assessment or treatment is remarkably slow when compared with the pace of technological development (Price et al. 2013). Although most existing DHTs are modified or enhanced digital versions of existing in-person behavioral interventions or bibliotherapeutic techniques (Marsch and Dallery 2012), adoption of DHTs will only become widespread as reputable studies demonstrate efficacy.

Unfortunately, many evaluations of DHTs to date have been flawed or limited only to certain populations. Recent reviews of computer-based interventions found significant methodological flaws in research designs, evaluations of treatment exposure and adherence, rates of follow-up assessment, and conformity to intention-to-treat principles (Kiluk et al. 2011). Furthermore, the well-designed trials solely assessing alcohol use typically have targeted young binge drinkers—a highly specific sample that under-represents the heterogeneity of the broader problem-drinking population.

DHTs discussed above that have been integrated into traditional alcohol and drug treatment with success, such as TES and A-CHESS, have used rigorous study designs and have dealt with more severely affected substance-using populations. However, it is important to note that many of the studies on DHT treatment integration have been conducted among polysubstance users, rather than alcohol users specifically, which limits generalizability to the latter population.

In the only review of mobile applications for alcohol use problems to date, Cohen and colleagues (2011) highlighted the dearth of outcomes and quality-control guidelines for alcohol intervention apps that are directly available to consumers. However, credible governmental Web sites, such as the Health Apps Library in the United Kingdom, may assist organizations in evaluating which DHTs are safest and best suited to their needs, even when a controlled trial has not been conducted on an application translated from the empirical literature. Also, in contrast to implementation of in-person, evidence-based treatments, which generally is slow going (Kumar et al. 2013), once a DHT is validated, it can be disseminated rapidly.

## Readying Organizations to Adopt DHTs

Once DHTs reach an acceptable level of validation, their successful integration into a treatment practice will depend on the staff’s preparedness and willingness to learn new technologies. Organizational and staff norms tend to reinforce the status quo rather than focusing on continuous quality improvement. Agencies should focus on integrating new procedures into existing workflows, creating new staff roles (e.g., project managers who will train staff and deal with resistance to or fear of integration), balancing a DHT’s financial costs against its potential rewards, and understanding how technology shifts certain roles and responsibilities. A flexible deployment model that focuses on the best uses of DHTs within an organization and harnesses the strengths of existing resources is a useful first step (Marsch and Gustafson 2013).

Even with organizational support and a technology project manager, there often are staff-related barriers to technology adoption. The most pressing staff concerns usually fall within the realms of time burden and level of comfort with the use of technology (Campbell et al. 2012). Understanding how to use the actual technology is one of the most common barriers to integration across all domains. For example, Kuhn and colleagues (2014) found that although all VA providers expressed fairly high interest in integrating a smartphone app into posttraumatic stress disorder treatment, younger clinicians and those with smartphones found the app more usable than older clinicians and those without smartphones. These variables predicted clinicians’ intentions to use the app in treatment. Integration requires an understanding of staff members’ degree of comfort with technology and the selection of appropriate training to increase staff confidence in navigating potentially foreign technologies.

Even when providers are trained and confident in the use of DHTs, new continuing-care applications in which providers view dashboards or are constantly on call increase rather than decrease staff burden by expecting staff to exceed their typical job responsibilities. Thus, managers should understand how any new technology will affect staff workload before adopting it. For example, Muench and colleagues (2013) found that although 80 percent of providers want to be alerted if their client is at risk of relapse, only 8 percent would want an immediate mobile alert. Most providers are interested in e-mails or phone messages on their work phones so as not to be on call at all times. Developing rotating staff or peer on-call procedures like those used in hospital settings can help. Another option involves using a graded alert system based on the overall risk of relapse, as is used by the A-CHESS system. Graded alerts can reduce unnecessary staff burden. When taken together, the emerging trend towards minimal long-term continuous contact interventions as a standard of care will not only require a shift in treatment models but also a change in policies, staff, procedures, and payment methods.

The elephant in the room, however, is the question of how these technologies will shift provider roles and responsibilities and alter current treatment models. No evidence exists that DHTs are better than in-person care, and evidence suggests that higher-severity populations do better with in-person care than with standalone DHTs for alcohol use. However, even the suggestion that an automated system may perform some aspect of someone’s job as well as or better than that person can be inherently demotivating. This is the case with some diagnostic and follow-up systems that use big data to make diagnoses (Graber and Mathew 2008) or predictive modeling to understand the change process (Chih et al. 2014). Similar to the way Amazon has changed the publishing and bookselling industry, DHTs will change how we provide substance abuse care. Amazon did not reduce the amount that people read, but it did change how they buy books, how books are published, and what the job landscape within the publishing world looks like. DHTs require a rethinking of how to provide care and offer the opportunity to improve service delivery in new and innovative ways. For example, some evidence suggests that Internet-based interventions with human support are equally effective when delivered by a technician versus a clinician. The finding highlights the need to understand how DHTs will affect current models of care (Titov et al. 2010).

## Addressing Client Concerns

Despite clients’ apparent enthusiasm for DHTs, they have concerns that will need to be addressed, particularly in the context of mobile monitoring systems. First and foremost is the Orwellian nature of real-world monitoring. Clients may not feel comfortable being continuously monitored outside of the treatment setting, and some will feel that it imposes on their freedom. Assuring clients that DHT communication and monitoring is an optional component of treatment will help to reduce this concern. Second, clients need to be trained in the use of these systems, since evidence shows that comfort with technology is a driver not only of provider use but also of client use (Ranney et al. 2012). Moreover, training needs to cover how to deal with emergencies and service outages beyond what simple consent procedures discuss. For example, training clients that DHTs are not designed to respond to emergencies, that outages in service are to be expected, and that they should not anticipate 24-hour-a-day communication will serve to create realistic expectations about these programs.

Among many low-income clients, another common problem occurs when they have their phone service turned on and off repeatedly for not paying their bills on time. In other cases, individuals may have temporary or disposable phones and therefore change their phone numbers often, creating discontinuity in service provision (McClure et al. 2013). In a methadone treatment text-messaging study (Muench et al. 2013), approximately 20 percent of participants had their phone service turned off at least once over the course of the 5-week study as a result of nonpayment (Muench et al. 2012). When their phones were eventually turned on again, clients received all of the messages they had missed over the stoppage period at once. Several individuals were also using family phones, raising unanticipated questions about confidentiality and privacy. This problem occurs across mobile interventions—whether they be smart-phone applications or text-messaging programs. Finally, DHTs that promise regular contact can fail because of programming and communication errors, which can become a source of stress for clients. Whereas an informed consent procedure may warn individuals of these lapses in communication, it may not prepare them for an instance in which they expect communication that never occurs.

Finally, smartphone technology presents challenges of its own. It is unfair and possibly unethical to integrate services into treatment programs that only a subset of clients will have the resources to access. The more expensive smart-phones have not yet saturated the mobile-phone market. Even when a client has a smartphone, problems arise with regard to interoperability between operating systems when using native applications (e.g., iOS vs. Android). Newer options that are device agnostic (i.e., that can be used on any mobile operating system), such as HTML5 applications, are becoming more commonplace, but still only account for about 30 percent of mobile development. These nuanced limitations of DHT integration are the rule rather than the exception. It is therefore imperative to any digital integration effort that treatment providers understand their population’s needs and constraints.

## Conclusions and Future Directions

Innovative DHTs that are now over 15 years old, such as the Drinker’s Check-Up, set the stage for the current proliferation of alcohol-related DHTs. Although research clearly emphasizes the benefits of DHTs to assess and intervene with individuals with low-severity alcohol problems, there is little evidence that standalone DHTs are helpful for those with more severe alcohol problems. Moreover, the current research suggests that standalone DHTs have limited long-term impact and high attrition rates, although there is evidence that adding mobile prompts improves the effectiveness of computer-based DHTs. The strongest evidence of efficacy to date supports DHTs that are included as part of in-person treatment as an adjunct or enhancement to current care. However, few treatment programs outside of research settings seem to be integrating assessment and intervention DHTs beyond electronic charting. This is attributed to a combination of tangible barriers, such as privacy and security concerns, organizational norms, unclear financial models, and lack of knowledge about the potential promises of DHTs beyond their pitfalls. Despite these uncertainties, more research now suggests that DHTs can at minimum be a helpful adjunct to various touch points in the treatment process. Treatment professionals can feel secure taking some initial steps into the world of DHTs without moving into the realm of equivocal efficacy. For example, one of the easiest methods to improve outcomes is to include digital client monitoring. This does not require mobile devices and tight security features but rather a weekly check-in at a kiosk behind the treatment center’s firewall. This could be a first step in using the power of DHTs to improve client outcomes without significant disruption of current models of care.

In the not-so-distant future, early identification of alcohol problems through predictive algorithms by supercomputers connected to a mobile application will warn individuals and providers of the likelihood of problem use long before problems start. These algorithms will alert individuals and counselors to relapse risk based on behavioral decisions that, although seemingly irrelevant at face value, in fact predict a lapse (Bekiroglu et al. 2013; Chih et al. 2014). For example, a recent simulation study of smokers revealed that machine learning applications can provide personalized JITAIs exclusively at times when support is most needed, which can reduce staff and client burden (Lagoa et al. 2014). Sensors on the mobile phone will be able to measure speech characteristics and gait of clients at risk of problematic alcohol use to trigger in-the-moment interventions that remind them to order a seltzer with lime rather than a beer. These technologies already exist and—once validated—will dramatically improve our ability to help individuals wanting to change their alcohol use. Mobile interventions might be especially powerful for individuals attempting to moderate their drinking. Unlike abstinence-oriented individuals for whom being in a high-risk situation would trigger an alert, individuals with moderation goals are often in high-risk situations and therefore require flexible adaptive drinking plans and methods to promote healthy drinking beyond stimulus control. This is where the nuances of smart systems will reveal their greatest benefits.

Clients and consumers are already embracing DHTs and creating a patient-centered health movement—a phenomenon that echoes the initial rise of 12-step treatments outside of medical institutions. This patient-centered movement empowers people to take control of their health. As evidenced throughout this paper, consumers will be the greatest beneficiaries of the digital revolution. In time, however, agencies and providers will experience significant benefits as well. Like all continuous quality-improvement systems, the integration of DHTs into treatment will be an iterative process that focuses on simultaneously maximizing outcomes and system harmony, which means that there will be many bumps in the road along the way. One question that requires an answer is how provider roles and responsibilities change as we integrate more DHTs into AUD treatment. However, as the research has repeatedly revealed, DHTs are most effective when combined with human support, reinforcing how providers will remain the foundation of care for those seeking help for their alcohol use for the foreseeable future.

## Figures and Tables

**Table:** DHT Examples, Including Some of the Most Common DHT Features, How They Can Be and Have Been Implemented in Alcohol Treatment Settings, and Some Basic Strengths of Each

DHT Feature	Examples of Use	Strengths
Treatment-based Digital Kiosk (Computer, Tablet, etc.)	Intake and follow-up assessments, psycho-education, virtual reality, digital enhancement, and replacement interventions.	“Captive audience” with provider contact to foster adherence and support. Behind firewall for enhanced security and data processing.
Client Computer/Mobile (General)	See above/below.	Distal assessment and intervention in one’s natural environment.
E-mail	Appointment reminders, Web links, group communications, natural-language processing.	Ubiquitous, inexpensive, high acceptability.
Text (including SMS, or short message service)	Appointment reminders, ecological momentary assessment, JITAIs (just-in-time adaptive interventions), Web links, natural-language processing.	Ubiquitous, real-time contact, inexpensive, high acceptability.
Camera/Video	Telehealth, modeling, distal environmental monitoring, journaling, exposure, ambient environmental analysis.	Ubiquitous, contextual, nonverbal, distal.
Sound	Speech analysis, environmental sound.	Ambient passive acoustic sensing, contextual environmental cues.
Geolocation	Trigger alerts, activity scheduling, positive activities, proximal social connections.	Objective location data, passive, social connectivity.
Accelerometer/Gyroscope	Activity assessment, behavioral activation, sleep, movement, side effects, intoxication.	Passive, objective, quantifiable, multiple existing systems.
Proximity Sensors	Proximal social monitoring, alerts.	Specific phones within private networks.
Mobile/Web Data Analytics	Everyday data pattern analysis, increases and decreases in social interaction, app usage.	Passive monitoring of secondary data, low invasiveness and battery drain.
Wireless Physiological Sensors: (e.g., Heart Rate Variability, Add-ons)	Physiological reaction and arousal, ability to predict outcome with objective data, relapse and side effects.	Objective data, physiological reactions outside of awareness, contextualized self-report.
